# Successful Extracorporeal Membrane Oxygenation After Incidental Azygos Vein Cannulation in a Neonate With Right-Sided Congenital Diaphragmatic Hernia Interruption of the Inferior Vena Cava and Azygos Continuation

**DOI:** 10.3389/fped.2019.00444

**Published:** 2019-10-25

**Authors:** Alessandra Mayer, Genny Raffaeli, Federico Schena, Valeria Parente, Gabriele Sorrentino, Francesco Macchini, Anna Maria Colli, Lucia Mauri, Simona Neri, Irene Borzani, Ernesto Leva, Fabio Mosca, Giacomo Cavallaro

**Affiliations:** ^1^NICU, Fondazione IRCCS Ca' Granda Ospedale Maggiore Policlinico, Milan, Italy; ^2^Betamed Perfusion Service, Rome, Italy; ^3^Department of Pediatric Surgery, Fondazione IRCCS Ca' Granda Ospedale Maggiore Policlinico, Milan, Italy; ^4^Cardiology Department, Fondazione IRCCS Ca' Granda Ospedale Maggiore Policlinico, Milan, Italy; ^5^Pediatric Anesthesiology and Intensive Care Unit, Department of Anesthesia and Critical Care, Fondazione IRCCS Ca' Granda Ospedale Maggiore Policlinico, Milan, Italy; ^6^Pediatric Radiology Unit, Fondazione IRCCS Ca' Granda Ospedale Maggiore Policlinico, Milan, Italy; ^7^Department of Clinical Sciences and Community Health, Università degli Studi di Milano, Milan, Italy

**Keywords:** ECMO, CDH, inferior vena cava, azygos, free hemoglobin

## Abstract

Incidental azygos vein cannulation has been reported in a few cases of neonatal extracorporeal membrane oxygenation (ECMO). This complication is described in the literature mainly in pathological conditions wherein increased central venous pressure dilates the superior vena cava (SVC), i.e., right congenital diaphragmatic hernia (CDH) or pulmonary hypertension. Azygos vein cannulation should always be suspected in cases of impaired venous return and circuit failure. Although rare, it hinders proper venous aspiration of the ECMO circuit and generally requires repositioning or replacement of the venous cannula or conversion to central cannulation. In this report, we describe a newborn with severe right CDH who required ECMO assistance, wherein incidental cannulation of the azygos vein resulted in successful functioning of the circuit because of the concomitant presence of isolated interruption of the inferior vena cava and azygos continuation. To the best of our knowledge, this is the first report of successful neonatal ECMO despite azygos vein cannulation in a patient with such rare physiology.

## Introduction

Extracorporeal membrane oxygenation (ECMO) is a type of extracorporeal life support that provides temporary support for patients with severe respiratory or cardiovascular failure refractory to conventional medical therapy (1). In the last several decades, medical advances in treating respiratory distress and pulmonary hypertension have progressively decreased the need for neonatal ECMO in respiratory failure (2). Currently, congenital diaphragmatic hernia (CDH) accounts for about one-third of all annual respiratory runs, especially when right-sided (3, 4).

Incidental azygos vein cannulation is a rare but potentially life-threatening complication described in a few reports of neonatal ECMO, mostly involving right-sided CDHs. To date, repositioning of the cannula is crucial to avoid circuit failure. This happens because the azygos-hemiazygos venous system usually provides collateral circulation between the superior vena cava (SVC) and inferior vena cava (IVC), collecting deoxygenated blood from the posterior chest and abdominal wall; it usually originates at the lumbar level from the union of the ascending lumbar vein and right subcostal vein, enters the thorax through the aortic hiatus, and then ascends in the posterior mediastinum before entering the SVC (5, 6). Interrupted IVC and azygos continuation is the most common venous anomaly involving the inferior caval vein and occurs in 0.3% of the general population and 0.6% of all patients with congenital heart disease. This kind of malformation is observed in heterotaxy syndromes, in particular, left atrial isomerism, also known as polysplenia syndrome due to the presence of multiple spleens (7, 8).

Here, we report a case of an ECMO successfully carried out despite cannula malposition in the azygos vein in a newborn with right-sided CDH, due to interrupted IVC and azygos continuation.

## Case Presentation

Ethics committee approval was not required for the description of the clinical case. The parents provided written informed consent for the publication of this case report in accordance with the Declaration of Helsinki. A 3,280-g male infant was born by planned cesarean section at 39 weeks of gestation after a pregnancy complicated at 30 weeks by the diagnosis of right-sided liver-up CDH.

At birth, the baby, whose APGAR score was 4 and 8 at 1 and 5 min, respectively, was electively intubated in the delivery room and ventilated with a fraction of inspired oxygen of 1.0. In the neonatal intensive care unit, the infant was initially administered high-frequency oscillatory ventilation with inhalation of nitric oxide and inotropic support (dopamine, dobutamine, hydrocortisone, and milrinone) because of hypotension and hemodynamic instability. Echocardiography confirmed severe pulmonary hypertension, left ventricular dysfunction, and no other congenital-associated anomalies. Despite aggressive medical management, severe hypoxemia, an oxygenation index >40, and mixed acidosis persisted. Therefore, on day of life (DOL) 1, the patient was promptly placed on veno-arterial ECMO (VA-ECMO). Surgical open cannulation of the neck vessels was performed, and cardiopulmonary bypass (Quadrox iD, Getinge®) was established from the right internal jugular vein (12 Fr Biomedicus, Medtronic®) to the right carotid artery (10 Fr Biomedicus, Medtronic®). A few hours later, surgical correction of CDH was performed, without surgical complications.

Nevertheless, from ECMO initiation, we encountered suboptimal venous drainage, with higher than expected suction pressures, based on the cannula pressure drop at different flow rates. Indeed, when attempting to increase the cardiac index to 150 mL/kg/min, we obtained increased drainage pressures (greater than −80 mmHg). First, an adequate intravascular volume status was guaranteed with crystalloid boluses, without improvement. Second, kinks or obstructions were excluded both along the ECMO venous line and venous cannula. Despite the position of the cannula appearing correct on the chest X-ray, the situation was highly suspicious for venous cannula malposition.

**Figure 1:**
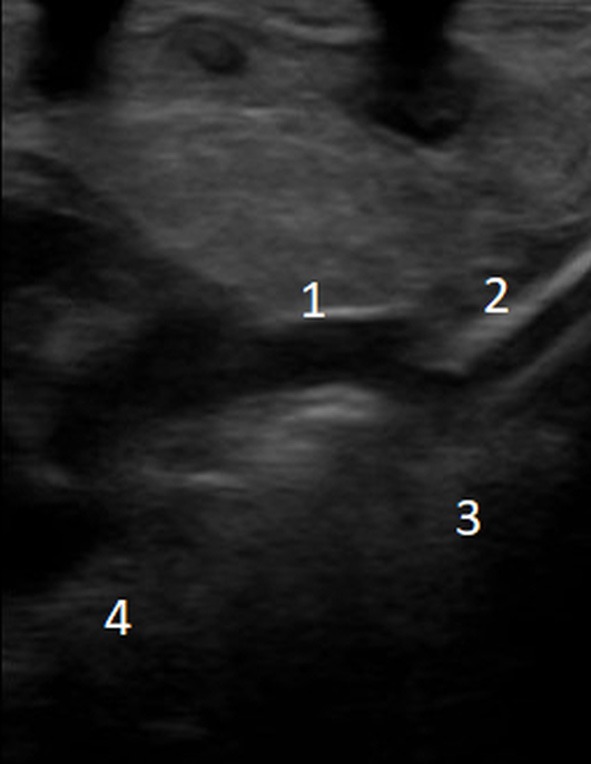
Echocardiographic evaluation of venous cannula position: (1) superior vena cava; (2) venous cannula; (3) azygos vein; (4) right atrium.

**Figure 2:**
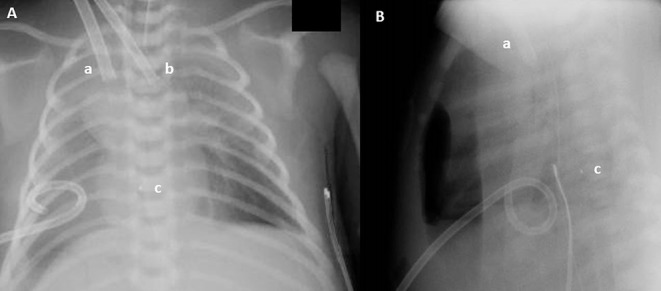
Anteroposterior **(A)** and lateral **(B)** chest X-ray: Drainage cannula (12 French) inserted in the jugular vein (a), inflow cannula (8 French) inserted in the carotid artery (b), and cannula tip (c). Even if in **(A)** the venous cannula seems to have a correct position, lateral chest X-ray reveals that the tip (c) of the venous cannula lies posteriorly to the heart.

A new ultrasound showed the venous cannula in the SVC adjacent to the confluence of the brachiocephalic veins with its tip protruding into the azygos vein (Figure 1). Interruption of the IVC and azygos continuation was then checked for and clearly shown. A lateral chest X-ray was also acquired, which revealed that the venous cannula was placed posteriorly to the cardiac silhouette, confirming azygos vein cannulation (Figure 2).

**Figure 3:**
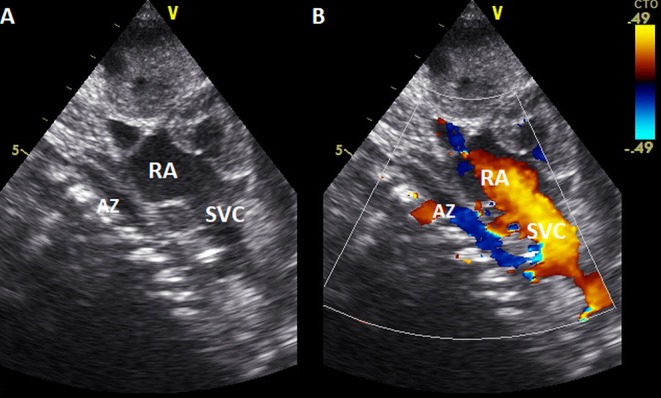
Echocardiography (2D and color Doppler, **A**, **B**, respectively) subcostal view, showing the ascending of azygos vein in the posterior mediastinum before entering in the superior vena cava. AZ, Azygos vein; RA, right atrium; SVC, superior vena cava.

**Figure 4:**
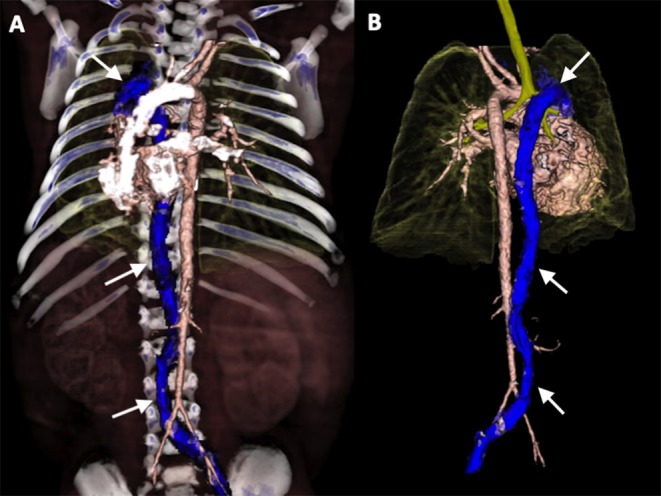
3D-volume rendering reconstructions of Computed Tomography Angiography (CTA) in anterior view **(A)** and posterior view **(B)**: demonstrating interrupted inferior vena cava with azygos continuation (arrows).

Despite the venous cannula malposition, an ECMO flow of at least 100 mL/kg/min was stably achieved throughout the ECMO course, and the patient remained clinically stable without requiring an increase in extracorporeal support. Moreover, hemolysis markers never reached critical levels, and the plasma free hemoglobin (PFHb) level decreased during the procedure from a maximum of 34.5–17.8 mg/dL. For these reasons, no action was deemed necessary, and the venous cannula was left in the azygos vein (Table 1). Serial cerebral ultrasound examinations were performed, and no cerebral bleeding was noted. On DOL 5, once the weaning trial was well-tolerated, the patient was successfully decannulated. New echocardiography was repeated with confirmation of interruption of the IVC and azygos continuation (Figure 3). On DOL 16, the infant was extubated and placed on non-invasive respiratory support. Computed tomography angiography confirmed the diagnosis of interrupted IVC and azygos continuation as an isolated anomaly (Figure 4). Brain magnetic resonance imaging excluded focal parenchymal lesions secondary to the ECMO procedure. The patient was finally discharged from the hospital with high-flow nasal cannulae at 2 months of age.

**Table 1:** Main respiratory and hemodynamic settings before, during, and after veno-arterial extracorporeal membrane oxygenation (ECMO).

**ECMO day**	**0**	**1**	**2**	**3**	**4**	**+1**
HR, bpm, mean (range)	140 (130–150)	151 (123–174)				126 (121–129)
Pre-ductal SpO_2_, %, mean (range)	82 (82–82)	95 (92–97)				95 (93–97)
Post-ductal SpO_2_, %, mean (range)	NA	96 (92–99)				NA
AP, mmHg, mean (range)	40 (35–45)	58 (41–71)				57 (50–69)
SvO_2_, mmHg, mean (range)	NA	84 (70–93)				NA
pH (mean ± SD)	7.22 (±0.13)	7.39 (±0.06)				7.46 (±0.05)
pO_2_, mmHg (mean ± SD)	36.45 (±0.78)	50.35 (±10.38)				67.53 (±2.26)
pCO_2_, mmHg (mean ± SD)	61.3 (±18.38)	46.22 (±8.06)				41.37 (±5.02)
Lactate, mmol/l (mean ± SD)	1.3 (± 0.28)	1.69 (±0.23)				0.93 (±0.12)
BE, mmol/l (mean ± SD)	−2.65 (±1.91)	2.76 (±1.62)				7.53 (±1.50)
Temperature, °C (mean ± SD)	36 (±1.34)	36.7 (±0.12)				36.4 (±0.36)
Cerebral rSO_2_ left, %, mean (range)	NA	80.00 (75–87)				NA
Cerebral rSO_2_ right, %, mean (range)	NA	78.25 (69–84)				NA
PFHb, mg/dl	NA	34.5	23.7	26.9	17.8	NA
**RESPIRATORY SETTINGS**						
Mode	HFO	PC/AC	PC/AC	PC/AC	PC/AC	PC/AC
FiO_2_	1	0.4	0.37	0.32	0.30	0.33
PIP, cmH_2_O	NA	25	25	27	25	30
PEEP, cmH_2_O	NA	7–9	7	7	6.5	6
Paw, cmH_2_O	15.5	11	10.6	11.7	10.5	12.3
RR/Hz	9	25	52	49	45	35
Vt (ml/kg)	NA	1.9	2.8	3.0	3.6	4.7
OI	42	NA	NA	NA	NA	<5
Surfactant BAL		X				
**CIRCULATORY SETTINGS**						
CI (ml/kg/min)	NA	98	103	106	80	NA
LPM pre-UF	NA	0.58	0.56	0.55	0.42	NA
LPM post-UF	NA	0.39	0.34	0.32	0.27	NA
RPM	NA	2,353	2,237	2,236	1,828	NA
FiO_2_	NA	0.4	0.37	0.26	0.21	NA
Sweep gas, l/min	NA	0.15	0.13	0.10	0.10	NA
P ven, mmHg	NA	−2	−3	2	0	NA
P int, mmHg	NA	152	129	136	107	NA
P art, mmHg	NA	145	124	134	126	NA
Delta P, mmHg	NA	7	3	2	2	NA
Dopamine (mcg/kg/min)	7.5	10	10	10	2.5	Stop
Dobutamine (mcg/kg/min)	5	5	7.5	5	2.5	Stop
Hydrocortisone (mg/kg/h)	0.1	0.3	0.3	0.3	0.3	0.3
Milrinone (mcg/kg/min)	0.75	0.75	0.75	0.75	0.75	0.75
**FLUID MANAGEMENT**						
Fluid overload (%)	NA	+2.2	−0.21	−1.5	−1.5	−1.5
Weight (g)	3,280	NA	NA	NA	NA	3,400
Urine output (cc/kg/h)	2.8	4.9	6.0	7.5	4.4	6.1
Ultrafiltration	NA	Off	On	Off	On	NA
Furosemide (mg/kg/24 h)	No	1.5	No	1	1	1
Albumine (g/kg/24 h)	No	No	2	2	No	No

*Trend of vital parameters, gas analysis main values, respiratory, and circulatory settings before ECMO (0), during ECMO (days 1–4), and after ECMO discontinuation (day +1). ECMO, extracorporeal membrane oxygenation; SD, standard deviation; HR, heart rate; SpO_*2*_, peripheral arterial oxygen saturation; AP, arterial pressure; SvO_*2*_, central venous oxygen saturation; BE, base excess; PFHb, plasma free hemoglobin; rSO_*2*_, regional oxygen saturation; pO_*2*_, partial pressure of oxygen; pCO_*2*_, partial pressure of carbon dioxide; FiO_*2*_, fraction of inspired oxygen; PC/AC, pressure controlled/assisted controlled; HFO, high frequency oscillatory ventilation; PIP, peak pressure; PEEP, positive end-expiratory pressure; Paw, mean airway pressure; RR, respiratory rate; Hz, hertz; Vt, tidal volume; OI, Oxygenation index; BAL, bronchoalveolar lavage; CI, cardiac index; LPM, liters per minute; UF, ultrafiltration; RPM, revolutions per minute; Fluid overload (%) = [(fluids IN–fluids OUT)/entry weight] × 100; h, hours*.

## Discussion

Azygos vein cannulation is a rare occurrence during the ECMO procedure. To the best of our knowledge, only a few neonatal cases have been previously described in the literature (7–11). Fisher et al. described two patients with right-sided CDH (9). In right-sided CDH, erroneous azygos vein cannulation can occur because of geometrical anomalies caused by right-sided CDH itself (9). Liver herniation in the right hemithorax causes IVC compression and subsequent azygos dilatation (9).

Moreover, a leftward mediastinal shift caused by right-sided lung hypoplasia alters the angle at the junction between the SVC and azygos vein, which becomes obtuse (9). All these anatomical modifications explain why patients with right-sided CDH can be more prone to azygos vein cannulation while being placed on ECMO (9). Interestingly, in both cases described by Fisher et al., the malposition of the venous cannula caused failure of ECMO support because of low blood flow. As the cannula position is a major determinant of the adequacy of ECMO, attempts to adjust venous cannula placement in the right atrium were required (9). Choi et al. described a similar case of azygos vein cannulation in a newborn with right-sided CDH in 2016; as previously described, repositioning of the cannula was needed to achieve adequate ECMO flow (10).

On the other hand, Byrnes et al. described a case of accidental azygos vein cannulation in a neonate with prenatal closure of the ductus arteriosus (11). This newborn presented with severe persistent pulmonary hypertension unresponsive to maximal medical therapy, mandating placement on VA-ECMO (11). Prenatal closure of the ductus arteriosus causes *in-utero* development of pulmonary hypertension, which leads to ventricular hypertrophy and right chamber dilation with progressive cardiac failure and fetal hydrops in the most severe cases (11). This condition, therefore, produces azygos vein dilation secondary to an elevation in SVC pressure, which in turn can facilitate azygos vein cannulation (11). Here again, venous cannula misplacement affected proper functioning of the ECMO circuit, dramatically limiting venous drainage, and ECMO flow to low levels. In this particular case, manipulation of the venous cannula was insufficient to restore adequate ECMO support, and replacement of the cannula over a guide wire was necessary (11).

Finally, in 2018, Runkel et al. reported a case of azygos vein cannulation in a neonate with persistent pulmonary hypertension that was promptly recognized and corrected by echocardiography during the cannulation procedure, avoiding subsequent reintervention (12).

When attempts to reposition the cannula are unsuccessful, conversion to central cannulation must be planned urgently, as described by Nosavan et al. (13).

In our case as well, peculiar anatomical and physiological features of right-sided CDH played a critical role in causing unintended azygos vein cannulation, but in this specific situation, with interruption of the IVC leading the whole lower systemic venous to return to the azygos vein itself, it allowed for good functioning of the circuit rather than representing a complication (9, 11). This is even more important if we consider that we could not choose or plan azygos cannulation because important displacement and compression of vascular structures by abdominal organs allowed the diagnosis to be made only postoperatively. Despite the greater length, course, and slightly smaller size of the azygos vein, compared to the IVC, we could still obtain adequate venous drainage and ECMO support, which was maintained between 90 and 120 mL/kg/min throughout the run, allowing for sufficient venous drainage. As evidence of this, the PFHb level never indicated the presence of critical hemolysis and decreased over time. Hemolysis is an intrinsic feature of all extracorporeal circuits and can be secondary to high negative pressure, clots within the circuit, or excessive pump speed (14, 15). In the adult, PFHb levels >50 mg/dL assessed 24 h post-ECMO initiation is considered an independent predictor of mortality (16). Recently, hemolysis has been associated with a ten-fold increase in mortality in pediatric patients with PFHb levels >30 mg/dL (17), while previously a six-fold increase in mortality was observed with PFHb levels >100 mg/dL in another pediatric cohort (18). In contrast, Dalton et al. did not identify an association between hemolysis and mortality, even if they reported higher PFHb values to contribute to renal failure (19).

Regarding the cannula position, chest X-ray is conventionally used to assess the adequacy of ECMO cannula placement through evaluation of the cannula tip, which is radiopaque.

Nevertheless, anteroposterior chest X-ray is often insufficient and can be misleading. In cases of azygos vein cannulation, lateral chest X-ray can be a valuable diagnostic tool, as it reveals the tip of the cannula lying posteriorly to the heart (9, 10). However, it is non-contributory in other kinds of cannula misplacement (i.e., coronary sinus cannulation).

In this specific case, lateral chest X-ray and two-dimensional echocardiography were superior in assessing correct cannula placement and can be used to confirm cannula position before the neck wound closure, in order to avoid subsequent cervical re-exploration for cannula manipulation or replacement (20).

Moreover, cannulation under echocardiographic guidance may prevent other kinds of complications, for instance, blood vessel perforation. In CDH, perforation of the SVC has been described as a possible complication due to the mediastinal shift that occurs in this situation, which exposes the SVC to a higher risk of trauma (21).

## Conclusion

In conclusion, every condition leading to elevation in SVC pressure and azygos vein dilation can cause its incidental cannulation when initiating ECMO support, particularly in patients with CDH and pulmonary hypertension, in whom this complication should always be considered depending on their peculiar physiology. In the presence of interrupted IVC and azygos continuation, ECMO is feasible despite cannulation of the azygos vein.

Intraoperative transthoracic echocardiography performed under sterile conditions during the cannulation procedure remains the cornerstone to prevent, identify, and correct cannula displacement. However, the decision to leave the cannula in an imperfect position may be the wisest if venous drainage remains adequate despite the malposition.

## Data Availability Statement

The datasets generated for this study are available on request to the corresponding author.

## Ethics Statement

Ethical review and approval was not required for the study on human participants in accordance with the local legislation and institutional requirements. Written informed consent to participate in this study was provided by the participants' legal guardian/next of kin.

## Author Contributions

AM, GC, GR, FS, AC, LM, and FMo contributed conception and design of the manuscript. VP, GS, and FMa collected the data retrospectively. AM, GR, FS, and GC wrote the first draft of the manuscript. All authors contributed to manuscript critical revision, read and approved the submitted version.

### Conflict of Interest

The authors declare that the research was conducted in the absence of any commercial or financial relationships that could be construed as a potential conflict of interest.
